# Super enhancers—Functional cores under the 3D genome

**DOI:** 10.1111/cpr.12970

**Published:** 2020-12-17

**Authors:** Juqing Zhang, Wei Yue, Yaqi Zhou, Mingzhi Liao, Xingqi Chen, Jinlian Hua

**Affiliations:** ^1^ College of Veterinary Medicine Shaanxi Centre of Stem Cells Engineering & Technology Northwest A&F University Yangling China; ^2^ College of Life Science Northwest A&F University Yangling China; ^3^ Department of Immunology, Genetics and Pathology Uppsala University Uppsala Sweden

**Keywords:** 3D genome, biological processes, phase‐separated condensates, super enhancer, transcription model

## Abstract

Complex biochemical reactions take place in the nucleus all the time. Transcription machines must follow the rules. The chromatin state, especially the three‐dimensional structure of the genome, plays an important role in gene regulation and expression. The super enhancers are important for defining cell identity in mammalian developmental processes and human diseases. It has been shown that the major components of transcriptional activation complexes are recruited by super enhancer to form phase‐separated condensates. We summarize the current knowledge about super enhancer in the 3D genome. Furthermore, a new related transcriptional regulation model from super enhancer is outlined to explain its role in the mammalian cell progress.

## Graphical abstract

As a regulatory element on the genome, the formation of super enhancers depends on specific chromatin states and spatial structures. A large number of regulatory molecules gather in the chromatin regions where the super enhancers are located. The molecules in these regions are very dense, forming huge transcriptional activation complexes, which interact with each other to construct phase‐separated condensates. Super enhancer‐mediated transcriptional activation depends on spatial proximity rather than a rigid bridge. There are molecular exchanges between the super enhancer‐associated condensate and the surroundings. Inactive Pol II is recruited and phosphorylated Pol II is excreted. Therefore, we propose a super enhancer‐mediated transcription mode to pave the way for future research.

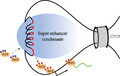

## 1 INTRODUCTION

Cell fate is precisely regulated in the developmental process and human disease. Since the discovery of Mendelian, gene regulation models are continuously developing.^1^, ^2^, ^3^, ^4^ In the beginning, the transcriptional models were linear.^5^, ^6^, ^7^, ^8^ In the 1980s, the first enhancer was identified which can increase gene expression by 200‐fold.^9^ With technological development, the study of chromatin is expanded into three dimensions (3D). Distal enhanced elements transmit the activation signal to the promoter by chromosome folding.^10^, ^11^ These advances further explained the accuracy of gene expression regulation, enriching theories of transcriptional models.

In 2013, Young and colleagues came up with the concept of the super enhancer (SE), as the region in the embryonic stem (ES) cells where Oct4, Sox2 and Nanog co‐bind,^12^, ^13^ with a huge region of the genome.^14^, ^15^, ^16^ Later, more and more biological macromolecules were found in the formation of SEs, and more biological functions were found to be controlled by them.^17^, ^18^, ^19^, ^20^, ^21^ Based on the characteristics reported previously, we describe that a SE is a cluster of enhancers with short spacing in the genome occupying more than 12.5 kb, having accessible chromatin and enrichment of transcriptional activators and core transcription factors.^22^, ^23^, ^24^, ^25^, ^26^, ^27^ Super enhancers are ubiquitous and have higher activation capacity than typical enhancers, which are sensitive to cell state.^28^, ^29^, ^30^, ^31^, ^32^ They are found in a variety of cell types in the body and span many species such as humans and mice. In addition, the SEs are also identified in domestic animals.

Moreover, the chromatin phase‐separated condensate is also detected at the locus of SEs, providing us with a new direction for research. There is an increasing amount of evidence suggesting that the transcriptional activation of SEs does not depend on rigid construction. Here, the structural and functional basis of SEs in the 3D genome is discussed. A new working model has been proposed and used to explain some biological progress.

## 2 SUPER ENHANCERS RELY ON DETERMINED CHROMATIN STATE

There are two basic conditions for gene activation, accessible chromatin and active transcription machines. In eukaryotes, the chromatin structure of the SE plays an important role.^33^

### 2.1 Histone modification and chromatin remodelling

**FIGURE 1:**
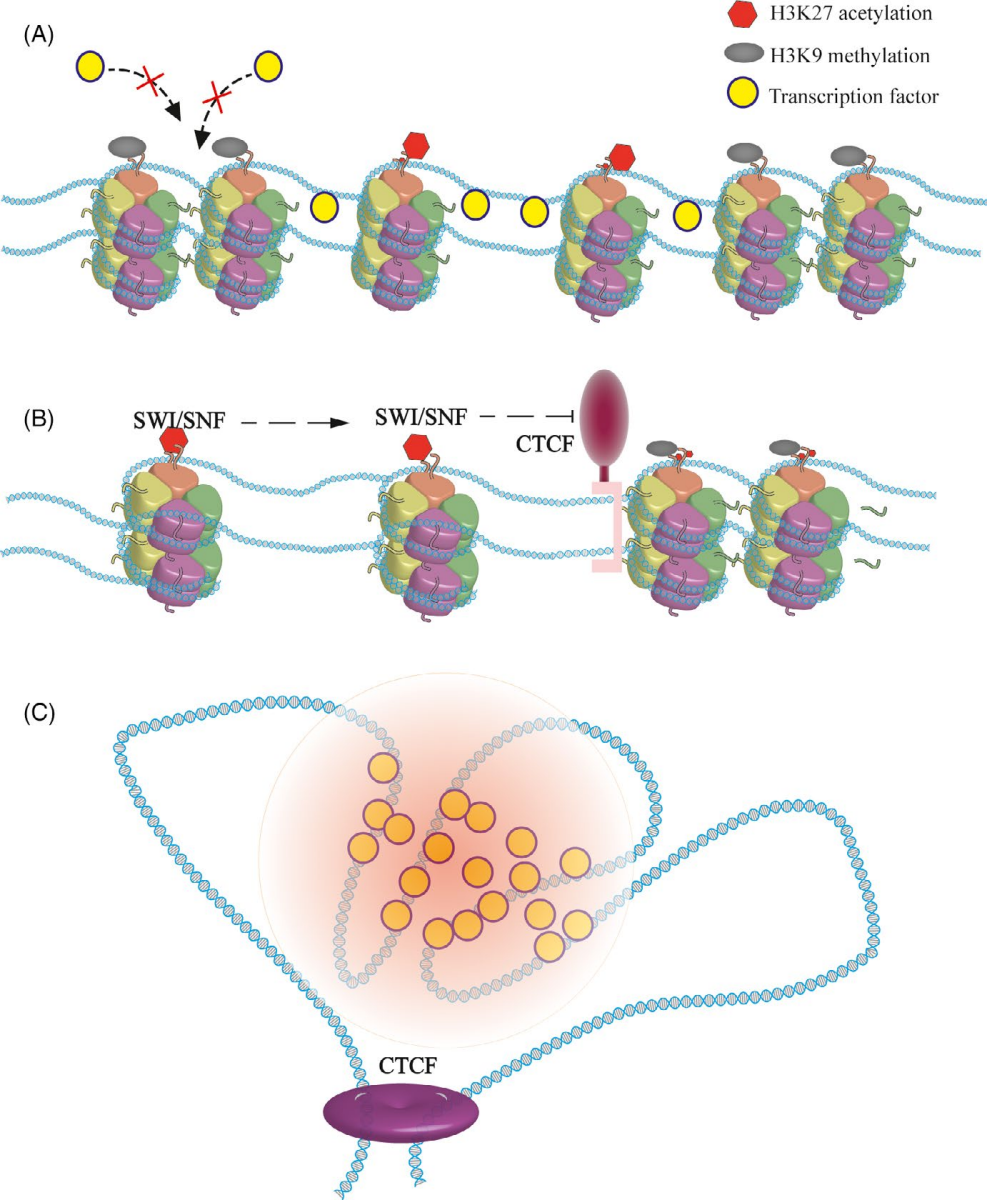
Super enhancers rely on a determined chromatin state. A, The maintenance of a super enhancer depends on epigenetic modifications. H3K27 acetylation loosens the chromatin and provides accessible transcription factor binding sites, which are essential for the function of the SE. In contrast, the H3K9 methylation region causing the chromatin to be denser and forming inert regions prevents the binding of active transcription machines. B, CTCF restricts the super enhancers in specific regions. With the help of chromatin remodelling complexes such as SWI/SNF, active chromatin modifications are prone to spreading, especially at the locus of SEs. The existence of architectural protein limits the SEs to a specific region, allowing for orderly gene regulation, and maintaining the conformation of the genome. C, Super enhancers rely on a determined chromatin state. The genome is divided into regions with the help of architectural proteins. In the regions where the SEs are located, the active transcription machine forms a phase‐separated condensate that maintains the precise regulation of genes

Young and colleagues found that H3 lysine 27 acetylation (H3K27ac) is the label for super ehnancers,^34^, ^35^ which makes chromatin structures looser, providing ideal sites for active transcription machine. In addition, master transcription factors tend to co‐bind at the SEs (Figure 1A).^36^, ^37^ In the case of mouse ES, the SE is occupied by the transcription factor Oct4. Once the sites of H3K27ac are changed, the binding of Oct4 was also changed.^38^ Furthermore, Histone H3 lysine 4 methylation is considered as a marker for the SEs as well.^39^, ^40^

Mohd‐Sarip proved that acetylated lysine residues are recognized by SWI/SNF, an important component of the chromatin remodelling complex.^41^ With the help of ATP, this complex can move labelled nucleosomes, forming exposed DNA.^42^, ^43^, ^44^ The depletion of SWI/SNF leads to the formation of inaccessible chromatin, resulting in the gene not being activated effectively even when the activators are present.

If chromatin is modified by inert epigenetic modification, such as H3K9 methylation, SEs will be destroyed. Indeed, H3K9 methylation can be recognized by heterochromatin protein 1 (HP1α), a common chromatin silence marker, which may cause the formation of heterochromatin.^45^, ^46^, ^47^, ^48^ Other histone modifications may also affect SEs. HDAC7 can regulate genes in breast cancer stem cells through SEs. When changed, the characters of SEs are shifted.^49^

With advances in microscopic imaging, chromatin phase‐separated condensates were observed in vitro.^50^, ^51^ Rosen and his colleagues found that specific histone modifications promote the formation of chromatin phase‐separated condensate. According to their report, when normal histone is acetylated, it destroys previous chromatin condensates. Nucleosomes that are nearby dissolve, forming an unstable state. After adding coactivators Brd4, which can be recruited by acetylated lysine, new condensates are constructed.^52^, ^53^ Super enhancers are very sensitive to histone modification. Excess or loss of modification may cause changes. With absolute quantification of architecture (AQuA‐HiChIP), Gryder discovered that rapid histone deacetylase inhibition resulted in abnormal contact, which destroyed the super enhancers.^54^ In summary, the formation of super enhancers in eukaryotes depends on the distribution of nucleosomes and the modification of histones.

### 2.2 DNA methylation and chromatin accessibility

The modification of DNA, especially DNA methylation, also has effects on the function of SEs. Generally, the GC ratio of SEs is much higher than that of typical enhancers. Hypomethylating agents, that is inhibitors are shown to reduce the activity of super enhancers.^55^ Among the different SEs, the GC ratio also varies. In 2020, Bell found that SEs are typified by distinctive CpG methylation dynamics.^56^ In the same locus, the DNA methylation level is different, and the activity of the SE is also different. Thus, at the same locus of the genome, different levels of DNA methylation lead to differences in SE activity between embryonic stem cells (ESCs) and epiblast stem cells (EpiSCs). Most of these affected genes are related to the naïve state.^56^ Moreover, Song reported that DNA methylation can regulate the level of H3K27ac through the balanced binding of DNA methyltransferase and transcription factors.^57^

The chromatin of the SE is highly accessible. Many studies have observed that the transcriptional regulatory regions identified by DNase hypersensitivity almost coincide with the locus of the super enhancer.^58^, ^59^, ^60^ The application of transposable‐accessible chromatin‐sequencing (ATAC‐Seq) provides further evidence.^33^ At the same time, histone‐less DNA is fragile and is prone to double‐strand breaks (DSBs). The DSB repair mechanism is closely related to SEs. If removed, the expression of SE‐associated genes decreased, further confirming this model.^61^

## 3 SUPER ENHANCERS RELY ON SPECIFIC TOPOLOGICALLY ASSOCIATED DOMAINS (TADS)

Previous studies have shown that chromatin modification at a site can alter the surrounding nucleosome dynamics, causing activation or silencing of nearby genes.^62^, ^63^, ^64^ It has also been proved that proximal enhancers activate promoters by diffusing. However, as a strong activator that plays a precise regulatory role, SEs must have individual working spaces. There is an abundance of data showing that SEs rely on specific TADs.^65^

Genomic loci are found to contact each other frequently. For instance, evaluating the three‐dimensional (3D) conformation of the HoxB locus in mouse ESCs revealed that, the architectural protein CTCF, essential for the formation of chromatin loops and enhancer‐promoter interactions, mediates the correct folding of the genome.^66^ The development of sequencing technology provides further evidence of the genome structure.^51^, ^67^, ^68^, ^69^ With Hi‐C, it is shown that TADs are fundamental elements of the eukaryotic genomic.^70^, ^71^, ^72^ Architectural proteins build chromatin to TADs and serve as the boundaries.^73^ Moreover, architectural proteins are extensively detected at the edge of the SEs.^73^, ^74^, ^75^, ^76^ Thus, it was determined that the CTCF is the boundary both for the TADs and the SEs, indicating the relationship between them (Figure 1B). In T‐cell acute lymphoblastic leukaemia (T‐ALL), when the CTCF‐mediated insulation is absent, the relevant TAD fused, and resulting in an abnormal contact between the SE and the promoter of MYC, leading to the up‐regulation of oncogenes.^77^ Differences in TADs can rewire interactions between the SE and the promoter.^78^, ^79^

Using high resolution in situ Hi‐C, researchers found that in mammals and insects the genome contains more TADs than we realized, most of which are smaller than what we detected with the previous Hi‐C.^80^ Different densities of chromatin interactions further affect the function of the SEs. In 2018, Yuan and colleagues observed that SEs are hierarchically organized.^81^ According to the frequency of chromatin interaction, SEs can be divided into hub and non‐hub enhancers. There are more frequent interactions in hub enhancers than in the non‐hub enhancers. The ablation of hub enhancers that have more influences resulted in profound defects in the cell state. Moreover, when using the CRISPR‐mediated chromosome modification to remove different small TADs in one SE, the expression levels of related genes were inconsistent.^82^, ^83^ These diverse and complex interactions are scattered within the SE providing more possibilities for the regulation of gene expression.

During genetic engineering operations, ectopic expression of cis‐regulatory elements and promoters can form TAD, but this is not a super enhancer. Similarly, in addition to maintaining the boundaries of the TADs, CTCF is also involved in the formation of sister chromatids. The functionality of the SE is included in the TADs. Super enhancers are cis‐regulatory elements that regulate gene expression. Indeed, they are a part of the genome. Super enhancers’ formation is based on specific genomic states (Figure 1C).^84^

## 4 SUPER ENHANCERS RECRUIT TRANSCRIPTIONAL ACTIVATION COMPLEXES TO WORK

**FIGURE 2:**
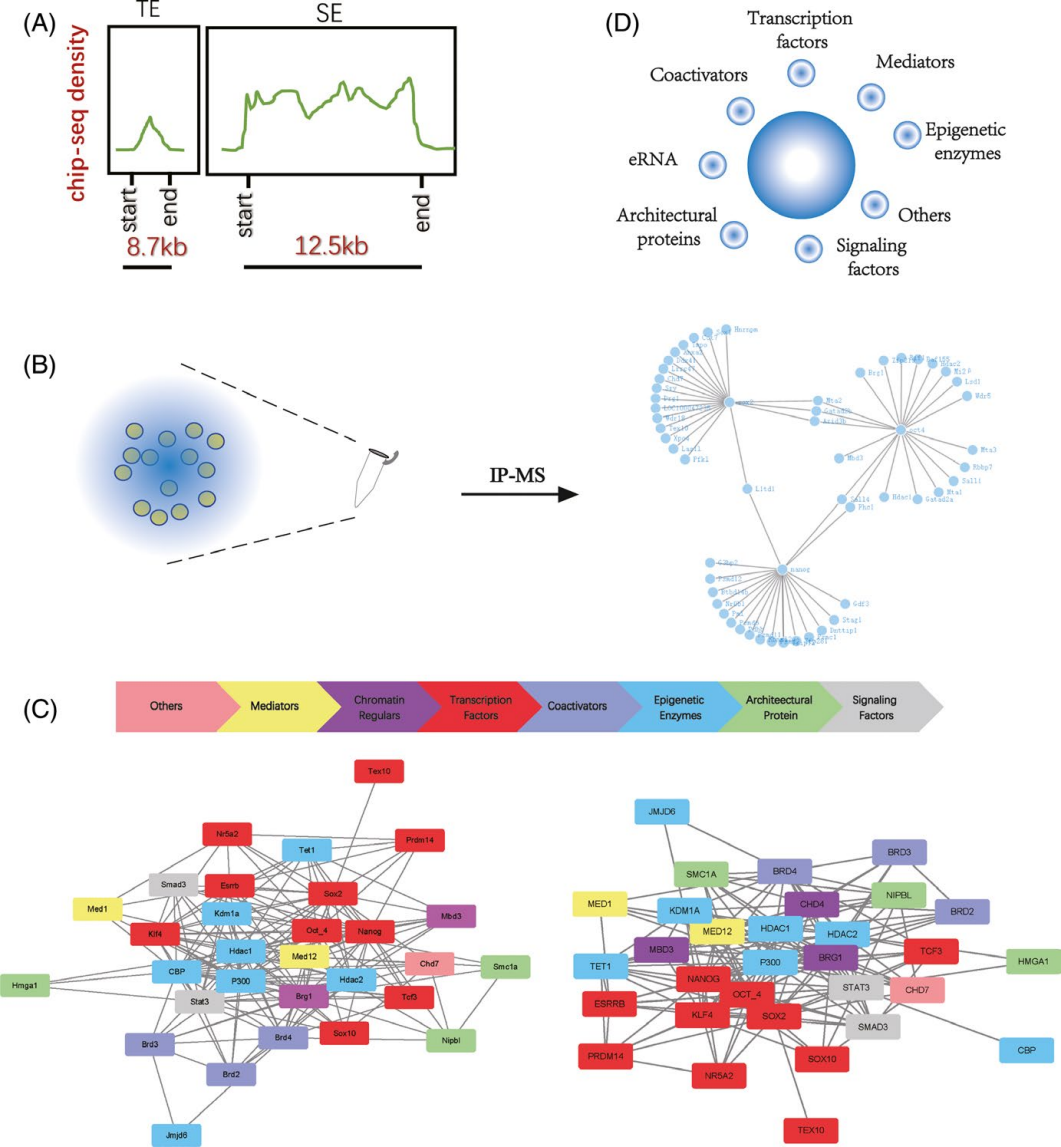
Super enhancers collect phase separation condensates. A, Based on previous studies, we have made a model diagram of the enrichment degree of transcription factors on SEs of mESCs. Comparison of the activation signals of a SE with a typical enhancer, the super enhancer occupies a larger logic of the genome which can collect more activation signals. B, By using previous IP‐MS data, we can identify the components collected by SEs. Here is the main composition collected by Oct4, Sox2 and Nanog in mESCs. A wide range of interactions can be observed among these factors. C, Network interactions of major proteins associated with super enhancers are reported in the literature. In both human and mouse pluripotent cells, the factors recruited by SEs are highly conserved. These factors can be divided into eight categories, and their interactions are extensive. D, Factors that are collected by SEs with extensive interactions can form phase‐separated condensates in physiological states. The main constituents of condensates are shown

Super enhancers have a stronger transcriptional activation capacity. The density of proteins close to the SEs is much higher in the genome, suggesting a frequent interaction (Figure 2A). A growing number of immunoprecipitation‐mass spectrometry (IP‐MS) data suggest that these macromolecules are linked to each other, forming huge complexes (Figure 2B). Specific chromatin conformation provides space for the binding of active molecules, and the execution of biological functions depends on the associated protein coordination.

### 4.1 Master transcription factors

Super enhancers were originally thought to be aggregates of core transcription factor binding motifs.^85^, ^86^, ^87^, ^88^ In mESCs, core transcription factors Oct4, Sox2 and Nanog are enriched in SEs to maintain the pluripotent network.^89^, ^90^ Of these, Oct4 was considered the most important. Under the naive state, genes associated with the SE are more sensitive to the inactivation of Oct4. Destruction of these SEs leads to the expression of genes similar to the depletion of Oct4.^15^ Later, this pattern was observed in various cell types. In 501mel melanoma cells, Sox10 was found to co‐locate with the H3K27ac labelled SEs. After the distribution of Sox10 was changed by inhibitors, the activity of melanocyte pigmentation changed.^91^

Analyses of the complexes in the region of SEs can help us find new master transcription factors. Combined with the IP‐MS data of Sox2 and related bioinformatics data, Ding et al, identified Tex10 as an important transcription factor for the establishment and maintenance of ESC.^92^ Using the same method, Shih‐Hwa and colleagues found Ash2l as recruiting activated complexes at SEs.^93^ To explore the Trophectoderm (TE) lineage development, Lee identified relevant SEs to look for TE specific transcription factors. More than 150 transcription factors have been identified, and 27 of them are highly correlated with gene transcription of TE.^85^ Collectively, the relationship between TFs and SEs helps us to understand gene regulation.

### 4.2 Mediators and coactivators

The mediators and coactivators are thought to bind together with enhancers.^94^ The concentration of mediators is higher than that of other regions which can also be used to identify SEs. In general, the mediator has 25 subunits in yeast and 33 in mammals which play an essential role in the regulation of Pol II.^95^, ^96^, ^97^, ^98^ In, 2019, Zamudio showed that mediators can bind to a variety of signalling molecules, assisting the cell type‐specific response.^99^ Different mediators have varying functions, some of which are found only in a particular lineage, while others are widely found in different lineages. In a certain state of cells, the SE loses its function when its core mediator is depleted.

Many studies have shown that the mediator has extensive interaction with coactivators.^100^, ^101^ The bromodomain and extra‐terminal domain (BET) protein family can recognize acetylated lysine residues. By mediating the formation of SEs, numerous studies have shown that Brd4 is related to SEs, can bind positive transcription elongation factor B (P‐TEFb) to promote transcriptional elongation.^102^, ^103^, ^104^ When cells are in the prime state of ESCs, the depletion of Brd4 leads to cell differentiation. When cells are in the naïve state, the combination of Brd4 and Tet1 maintains the pluripotency of the cells.^105^ Further research has shown that JQ1, a brd4‐specific inhibitor, can break SEs of cancer stem cells, becoming a new target for cancer.^106^ Furthermore, mediators and coactivators take part in the formation of the SE‐associated activation complex.

### 4.3 Chromatin regulars

It is shown that epigenetic enzymes are present in the chromatin complex.^21^, ^107^, ^108^, ^109^ CBP/P300, the main histone acetylase, has been shown to be a companion to the SE. Histone demethylase is no exception. In 2019, Wong et al reported that the histone demethylase JMJD6 can form protein complexes with BRD4, regulating the proliferation of neuroblastoma cells.^110^ Tet1, an extensive and powerful DNA demethylase, is also extensively involved in the regulatory network. In the process of mouse reprogramming, Tet1 was proved to be able to replace Oct4, completing the construction of ESCs related super enhancers.^111^ In addition, chromatin kinetics‐related molecules are also associated with SEs, such as Brg1, the most famous component of chromatin remoulding complexes. Meanwhile, other elements have been found such as the architectural protein and cohesion.^112^ Moreover, high mobility group proteins like HMGA1 are needed to maintain the enhancer substructures.

### 4.4 RNAs

In addition to proteins, RNAs also participate in the formation of SE‐associated complex. Long non‐coding RNA (lncRNA) is not distributed randomly in the genome but has an obvious overlap with the super enhancer.^113^ Also, Rothschild et al identified a strand of lncRNA that takes part in the SEs’ formation, without which, gene expression in B cells was blocked.^114^ Justifiably, enhancer RNA(eRNAs) cannot be absent in the SE, as they assist the transcription‐activated complexes to perform their duties.^115^ The eRNAs are synthesized on enhancers occupied by the Tet.^116^ During the differentiation of skeletal myoblast, seRNA‐1 and −2 generated by the SE drives myogenic differentiation.^117^ In Banani's study, eRNA acted as a scaffold for the formation of a protein‐nucleic acid complex.^118^ With Ric‐seq, Cai et al further provided evidence that RNAs and their binding proteins participate in the construction of 3D chromatin structures. Moreover, they also mediate the transmission of Pol II activated by the SE.^119^

### 4.5 Others

It has been reported that more and more molecules are components of SEs. Because of the large volume and high degree of integration in the SE‐related complex, almost all molecules that have similar locations are inevitably included. The nuclear matrix protein SAFA (also known as HnRNPU) activates immune‐related SEs to establish a cellular antiviral defence line after virus invasion.^120^ Scientists also found that a large number of signalling factors for the WNT, TGF‐b and JAK/STAT pathways also enter and concentrate in the complex at super enhancers.^121^, ^122^, ^123^

Due to the limitations in technology, it is not possible to enrich all components collected by SEs through experiments as a result of one or a few factors, but the increasing number of different data indicates that more and more molecules may be involved in this biological process (Figure 2C). These molecules interact to form complexes that support the function of the super enhancer.

## 5 PHASE‐SEPARATED CONDENSATES ARE FORMED BY SUPER ENHANCER‐ASSOCIATED COMPLEXES

The transcription‐activated complexes recruited by the SEs, as the very large functional polymers that have about 10‐fold molecular density than the typical enhancer, need a stable structure to maintain their conformation in a physiological state. Advances in biochemistry have often led to alternative theories. The discovery of the chromatin undergoing liquid‐liquid phase separation (LLPS) under physiologic conditions provides us with new insights.^124^ A series of recent developments indicate that the components of transcriptional activation complexes recruited by SEs take part in the formation of this condensate.^125^

In 2018, a report by Sabari showed that MED1 and Brd4 were important components of phase‐separated condensates.^126^ They share the same location and change rapidly after the environment of physiological salt is destroyed. Under the confocal microscopy, it was shown that phase‐separated condensates divide cells into small compartments, which are immiscible with unmodified chromatin droplets, like small membrane‐free organelles.^127^ Several parallel studies have also supported this theory. Scientists further discovered that the mediator and coactivator interact with other components through their intrinsically disordered regions (IDRs).^126^ According to Staby's study, disordered protein intrinsic disorders (ID) of transcription factors itself mediate many aggregations.^128^ Through several specific examples, Liu further elaborated on the contribution of this exquisite structure.^129^ In addition, Benjamin further explored ID mediated interactions and found that signalling factors could also take part in the formation of phase separation condensates through IDRs. On the other hand, from the perspective of energy, the SEs release more entropy by agglutination of more elements at higher density through a wide range of interactions, which is more trend for the construction of phase‐separated condensates.^130^

Of course, there is an extensive molecular exchange between the phase‐separated condensates and their environment. Young and colleagues proved that hypophosphorylated Pol II entered the mediator condensates preferentially through the IDR at the C‐terminal domain of the large subunit. Once phosphorylated by cyclin‐dependent kinases (CDKs), this incorporation was reduced and Pol II was released as a result of transcriptional activity.^131^ The processed Pol II is further recruited by RNA‐binding proteins close to the promoter of downstream genes to realize the transmission of the activation signal.^119^ Regulatory molecules constructing phase‐separated condensates to form micro‐organelles, provide a physical basis for the precise regulation mediated by SEs (Figure 2D).

## 6 SUPER ENHANCERS PERFORM TRANSCRIPTIONAL ACTIVATION THROUGH SPATIAL PROXIMITY RATHER THAN A RIGID BRIDGE

Scientists have shown that the workspace of the SE is a loop.^132^, ^133^, ^134^ Previous studies have hypothesized that the SE acts as a 3D structural architecture of cells recruiting a variety of factors to form a large complex that achieves physical contact with the promoter, that is, there are rigid physical bridges. This theory seems to be logical. An extensive amount of Chip‐seq data also seems to support this point.

**FIGURE 3:**
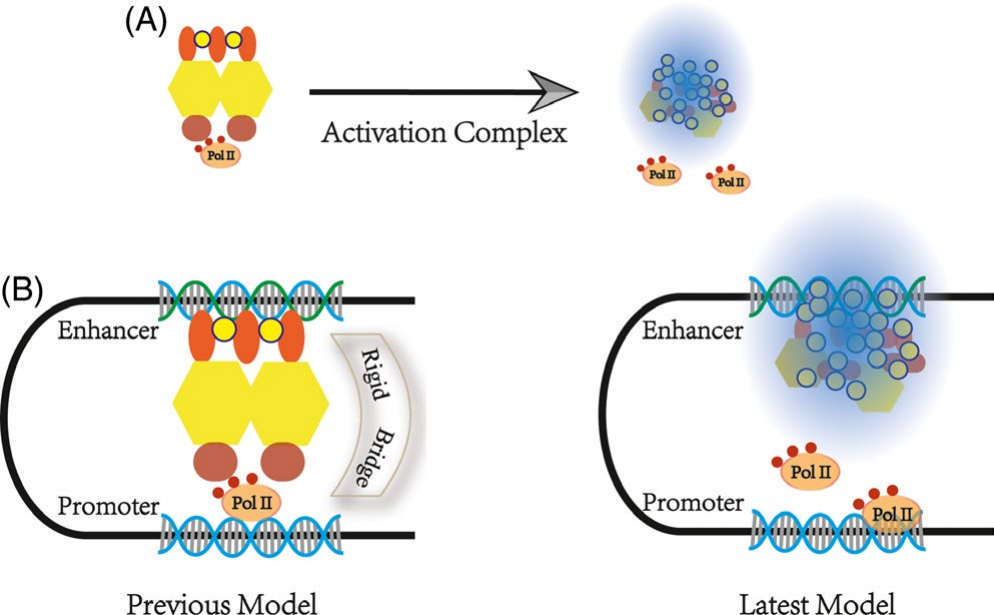
Enhancer‐promoter interaction model. A, The transcriptional activation complex recruited by the super enhancer is not rigid but exists as a condensate. B, Previous theories have suggested that there is a rigid bridge between enhancers and promoters. Recent studies have shown that SEs send activation signals to downstream genes through spatial proximity

However, this theory cannot explain the formation of chromatin phase‐separated condensate. From another point of view, the length of time that transcription factors and PolII remained on the chromatin was measured in seconds, using fluorescence recovery after photobleaching (FRAP) and single‐molecule experiments.^135^, ^136^ This temporal effect is insufficient to maintain the stability of the bridge structure. Using high‐resolution Hi‐C, Suhas SP Rao found that mammalian cells contained more transcription loops than previously reported. It was found that 90% of these transcription loops are associated with CTCF.^137^ In 2019, Khattabi described a new transcriptional activation hypothesis.^138^ With different degron systems, he observed that acute depletion of mediators and Pol II has little impact on P‐E contacts whereas the cohesin depletion does.^132^, ^139^ New technologies can usually explain old disputes; thus, the transcription activate machine comes into play when the enhancer comes into contact with the promoter by CRISPR/Cas9.^140^, ^141^ Architectural molecules bring the chromatin closer and form a stable conformation, providing a spatial basis for SEs.^142^ Above all, SEs that perform transcriptional activation do not depend on a rigid bridge but spatial proximity (Figure 3A and B).^143^, ^144^

## 7 SUPER ENHANCER—A NEW MODEL OF A TRANSCRIPTION MACHINE

**FIGURE 4:**
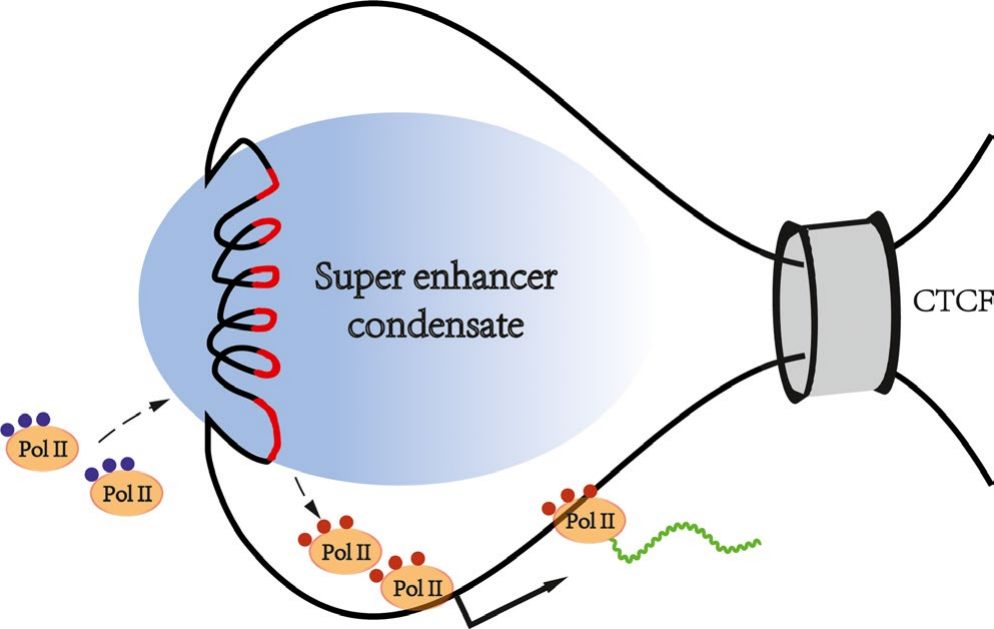
Super enhancer‐related transcriptional regulation model. With the help of architectural proteins, the chromatin structure is determined. The inactive transcription machine is recruited and activated by SE‐associated condensates. Mature transcription machines are then released and recruited to downstream genes

Based on previous publications, we propose a SE‐mediated transcription mode to pave the way for future research (Figure 4). First, as a regulatory element on the genome, the formation of SEs depends on specific chromatin states and spatial structures. Second, many regulatory molecules gather in the chromatin regions where the SEs are located. The molecules in these regions are very dense, forming huge transcriptional activation complexes, which interact with each other to construct phase‐separated condensates. Third, SE‐mediated transcriptional activation depends on spatial proximity rather than a rigid bridge. There are molecular exchanges between the super enhancer‐associated condensate and the surroundings. Inactive Pol II is recruited, and phosphorylated Pol II is excreted. High‐density biological macromolecules combine in an orderly fashion at the SE locus, forming membrane‐free organelles by chromatin phase separation, thus, ensuring the precise regulation of genes.

## 8 SUPER ENHANCERS REGULATE BIOLOGICAL PROCESSES

A gene can express in different cells, playing different roles with different conditions of chromatin.^145^ With an approach called clustering of genomic regions analysis method (CREAM), we can identify that the specific combination of cis‐regulatory elements determines the state of the cell.^146^ Although SEs can achieve a level of gene activation that is about 26 times that of typical enhancers, the activity of a single SE does not determine the fate of an entire cell. The fate of the cell depends on the combination of all SEs. Many SEs can exist in multiple lineages. For example, during the differentiation of ESCs to EpiSCs, some SEs can be detected continuously.^56^ With the same SE, different transcription factors can lead to different gene expression. Even with the same SE and the same transcription factors, different ligands also lead to changes. With nerve cells, LUSC, instead of P63, binds to SOX2, causing the cells to become cancerous.^147^ Minor differences in SEs can create a different landscape of gene expression. In pancreatic adenocarcinoma (PAAD), combined with transcriptome data, 169 genes were found to be associated with the copy number variations (CNVs) of elements in a super enhancer.^148^

Super enhancers are involved in many biological processes. The overactivation of proto‐oncogene is mostly related to the abnormality of SEs. The rapid rate of cell replication further accelerates the disordered assembly of chromatin.^149^ The abnormal fusion of the super enhancer's locus with other regions of the genome in cancer cells causes abnormal activation of proto‐oncogenes making it difficult to cure.^150^ The fate of the cell is dynamic, implying that there are also dynamic changes in the SEs. Nishida et al found that the high expression of inflammation‐related genes is turned on by epigenetic changes, especially the construction of SEs. Inhibition of the associated SEs can reduce this response.^151^

In vitro, the transformation of cell fate has also been shown to be associated with SEs. During the somatic cell reprogramming, histone modification‐related enzymes are recruited by core transcription factors and are pulled to the SEs’ region. As a result, the old SEs were broken. In the presence of Myc, chromatin becomes looser and the region of new SEs becomes exposed. Initially, established SEs began to play a weak role.^152^ After a brief intermediate state, a new cellular state is established.^153^ More interesting, only a few lucky cells can be completely reprogrammed. Some cells are only partially reprogrammed due to the incompletely established SEs. As reported in 2014, cells, which undergo an intermediate cell state with incomplete ES cell characteristics named F‐Class, were reprogrammed completely after adding the histone acetylase inhibitors, which further confirmed this theory.^154^ Of course, we can believe this transformation is widespread. As an important cell identification element, SEs are widely involved in biological processes.

## 9 PROSPECT

Since the establishment of molecular biology, scientists have long sought the rules governing molecules in the nucleus. It is generally accepted that gene activation requires two basic conditions, open chromatin and active transcription machinery. More and more researchers have devoted the transition model through the latest technology.^19^, ^131^ However, due to the differences in their research field, their results are fragmented. In this review, we summarize these studies and believe that the function of SEs depends on specific chromatin states. Architectural protein assemble chromatin into a precise 3D structure through extensive interactions. Also, the formation of SEs with the help of TADs provides the structural basis for the recruitment of transcription activate complexes. Moreover, these complexes are organized as a phase‐separated condensate, forming membrane‐free organelles that can regulate gene expression more precisely.

Furthermore, we optimized knowledge related to SEs. Previous theories have suggested that there are rigid physical connections between SEs and promoters. This structure acts as a bridge, sending activation signals downstream. However, this contradicts the latest progress in molecular biology. We modified the original model and propose that the SE activates downstream genes through spatial proximity. According to our model and previous research, the pattern of how SEs can regulate biological processes was also put forward in order to provide references for the following research.

More systematic analysis through multiple omics can help us grasp the main contradiction. The fate of cells is determined by core transcription factors. As a major binding region of the core transcription factors, SEs play an important role in determining cell identity. The expression of a gene is regulated by many molecules at the same time. Core transcription factors can be used to define SEs. Conversely, SEs can also be used to identify undefined core transcription factors, enriching the knowledge of transcription regulation.

The research on SEs can also solve problems in economic production and human life. In large animals, for example, understanding muscle‐specific SEs can help us to screen and breed high‐value livestock. Hearts from genetically modified pigs could be transplanted into baboons and that can be kept alive.^155^ However, until now, the true pig embryonic stem cells have not been established.^156^ Production of multigene‐edited pigs depends on repeated somatic cell nuclear transfers or inefficient microinjections. Understanding the SEs of pig embryos can help speed up this process. Moreover, SEs also have great potential in the immune system. Suppressing the activation can lead to abnormal immune system responses. Because of the powerful gene activation capabilities, tumours can occur once super enhancers interact with oncogenes. As the SE inhibitor, JQ1 can inhibit various types of cancers.

From the foregoing discussion, a basic trend emerges. The study of cell fate determination is becoming more and more systematic and refined. Systematic analysis allows us to better understand the state of the cell at a macro level, and refined regulatory mechanisms are essential to increase knowledge about the genome. As the functional core obtained from multiple omics, SEs must play more roles in cell processes. Understanding related regulatory mechanisms associated with SEs could help us solve more biological problems. The study of SEs will bring another leap forward in life science.

## CONFLICT OF INTEREST

All authors have reviewed the final version of the manuscript and given approval for its publication. This manuscript has not been published, in whole or in part, and is not under consideration for publication elsewhere. The authors have no conflicts of interest to declare.

## AUTHOR CONTRIBUTIONS

Juqing Zhang and Jinlian Hua designed the study and wrote the manuscript. Mingzhi Liao and Yaqi Zhou analysed the data. Wei Yue and Xingqi Chen made instructions and corrections to the language and content of the article.

## Data Availability

The data supporting the findings of this study are available from the corresponding author upon reasonable request.
